# Biological Filtering and Substrate Promiscuity Prediction for Annotating Untargeted Metabolomics

**DOI:** 10.3390/metabo10040160

**Published:** 2020-04-21

**Authors:** Neda Hassanpour, Nicholas Alden, Rani Menon, Arul Jayaraman, Kyongbum Lee, Soha Hassoun

**Affiliations:** 1 Department of Computer Science, Tufts University, Medford, MA 02421, USA; neda.hassanpour@tufts.edu; 2 Department of Chemical and Biological Engineering, Tufts University, Medford, MA 02421, USA; Nicholas.Alden@tufts.edu (N.A.); kyongbum.lee@tufts.edu (K.L.); 3 Department of Chemical Engineering, Texas A&M, College Station, TX 77843, USA; rmenon@mail.che.tamu.edu (R.M.); arulj@mail.che.tamu.edu (A.J.)

**Keywords:** metabolomics, metabolite annotation, enzyme promiscuity, extended metabolic models

## Abstract

Mass spectrometry coupled with chromatography separation techniques provides a powerful platform for untargeted metabolomics. Determining the chemical identities of detected compounds however remains a major challenge. Here, we present a novel computational workflow, termed extended metabolic model filtering (EMMF), that aims to engineer a candidate set, a listing of putative chemical identities to be used during annotation, through an extended metabolic model (EMM). An EMM includes not only canonical substrates and products of enzymes already cataloged in a database through a reference metabolic model, but also metabolites that can form due to substrate promiscuity. EMMF aims to strike a balance between discovering previously uncharacterized metabolites and the computational burden of annotation. EMMF was applied to untargeted LC–MS data collected from cultures of Chinese hamster ovary (CHO) cells and murine cecal microbiota. EMM metabolites matched, on average, to 23.92% of measured masses, providing a > 7-fold increase in the candidate set size when compared to a reference metabolic model. Many metabolites suggested by EMMF are not catalogued in PubChem. For the CHO cell, we experimentally confirmed the presence of 4-hydroxyphenyllactate, a metabolite predicted by EMMF that has not been previously documented as part of the CHO cell metabolic model.

## 1. Introduction

Metabolomics is an expanding field of research that involves the characterization of small molecules in cells, tissues, and other biological systems. Metabolites are direct products of enzymatic reactions that provide a functional readout of cellular state [1,2]. Compared to genes and proteins that are regulated and post-translationally modified, respectively, metabolites are most predictive of the phenotype [3]. Metabolomics now plays a critical role in many fields including drug discovery and precision medicine, nutritional analysis, and in examining environmental responses. Importantly, the ability to collect measurements on the metabolome using untargeted metabolomics, where thousands of features within the sample under study are measured and annotated with chemical identities, promises to broadly profile the metabolome and revolutionize phenotyping and biological discoveries.

Mass spectrometry (MS) techniques coupled with liquid or gas chromatography separation techniques, LC–MS or GC–MS, respectively, have become standard analytical platforms for untargeted metabolomics [4]. The LC or GC step aims to separate compounds within the sample, whereas the MS step ionizes, fragments, and detects a fragmentation pattern. There are now techniques for data processing (e.g., peak picking, missing value imputation, and adduct and degenerative feature removal). These tools convert raw MS data into features. Each feature corresponds to an ionized chemical compound, and is characterized using a spectral signature, comprising a chromatographic retention time (RT) paired with mass-to-charge ratio (*m*/*z*) and relative intensities for the parent compound and its fragments. 

Interpretation of metabolomics data is facilitated by assigning putative chemical identities to the features. Relying on the mass of the ionized parent compound for annotation is problematic, as a particular mass may be associated with many chemical formulas (e.g., there are 10,132 known molecular structures in PubChem [5] that are associated with C_20_H_22_N_2_O_4_) [6]. The spectra of detected compounds can be matched against those within an in-house library generated using the same instrument and method as the samples. However, this is impractical due to the large number of compounds detected in an untargeted MS experiment. Instead, feature annotation typically relies on libraries in reference databases. The two largest spectral databases in terms of number of unique compounds, METLIN [7] and NIST [8], cover only a small number of compounds when compared to the millions of compounds catalogued in PubChem. Due to these limitations, the annotation rate, which we define as the fraction of features annotated with a putative chemical identity, using in-house or spectral databases is typically low. The maximum annotation rate across several metabolomics studies that we surveyed was 16%, but averaged only 7.26% [9,10,11,12,13,14,15].

In recent years, computational tools have become available to recommend a ranked list of chemical structures that best explain a spectral signature. This ranked list is selected amongst a pre-specified candidate set, a listing of metabolites with formula weights that match the measured masses of parent compounds in the sample. Earlier tools used rule-based approaches to generate fragmentation patterns of candidate metabolites, e.g., [16]. Subsequent efforts introduced combinatorial enumeration methods [17,18,19] and machine-learning algorithms. For example, CFM-ID [20] uses the candidate set to create a probabilistic model of collision-induced fragmentation process. The model is then used to predict a fragmentation pattern for a given compound. CSI:FingerID [21] first predicts a fragmentation tree based on a spectral signature [22]. CSI:FingerID then uses multiple-kernel learning [23] and support vector machines to predict fragmentation tree properties, which are searched against fragmentation tree properties of compounds in a molecular structure database. CSI:FingerID, as well as subsequent updates within SIRIUS 4 [24], outperforms other tools [17,20,25,26,27] in terms of accuracy. Despite progress, however, annotation runtimes are costly [21], and are dependent on the size of the candidate set. Hence, evaluating candidate sets from large compound databases such as PubChem and ChemSpider remain problematic. 

We propose a novel annotation workflow for untargeted metabolomics that addresses current limitations regarding spectral database coverage and computational cost of annotation. The goal of this workflow is to engineer a candidate set that can be used for putative identification using database searches or other annotation tools. The key step is to filter the detected masses through a metabolic model that we call an extended metabolic model (EMM). An EMM includes not only the defined substrates and products of enzymes cataloged for the organism(s) associated with the sample, but also additional metabolites reflecting the potential for promiscuous enzymatic activities. The central premise is that an EMM can be used to define a candidate set that is more comprehensive than a standard genome-scale metabolic model, but still enforces a degree of specificity for the system of interest. Our workflow, termed EMMF (EMM-based filtering), broadens the search space for annotation beyond compounds in a reference metabolic model assembled from catalog definitions of enzymatic reactions, thus enhancing discovery while avoiding the computational cost of analyzing every compound in large chemical structural databases. We demonstrated the utility of EMMF on untargeted LC–MS data from cultures of Chinese hamster ovary (CHO) cells and bacterial isolates from murine cecum. We compared the candidate sets from reference metabolic models, EMMs, and a large structural database (PubChem). EMMF suggested biologically relevant chemical identities for almost a quarter of measured features, providing a > 7-fold increase in the candidate set size when compared to using a reference metabolic model. Importantly, EMMF allowed the discovery of novel relevant putative identities that are not currently catalogued in PubChem. Targeted LC–MS experiments confirmed the presence of a predicted CHO cell metabolite that had previously not been cataloged as a Chinese hamster enzyme substrate or product.

## 2. Methods

**Figure 1:**
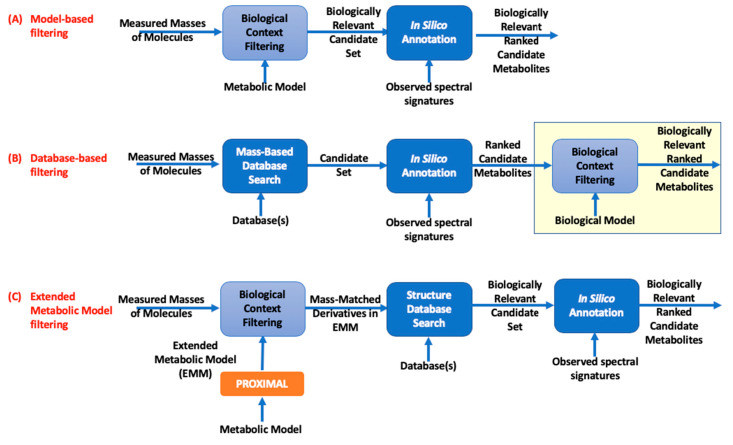
Comparison between annotation workflows. The candidate set for annotation is derived by filtering the measured masses based on: (**A**) the metabolic model, (**B**) databases, and (**C**) extended metabolic model (EMM). The candidate sets in (**A**) and (**C**) are biologically relevant, while candidates in (**B**) prior to filtering may not all be biologically relevant.

To describe and evaluate the EMMF workflow, we presented it alongside two other annotation workflows (Figure 1). In describing the workflows, “annotation” refers to the use of any computational annotation tools. A model-based annotation workflow (Figure 1A) consists of filtering masses of measured metabolites against those expected in the sample on the basis of a metabolic model that is built from a reference genome (or set of reference genomes). Model metabolites with exact masses that match, within a small error, to measured masses are designated as the candidate set. The candidate set is then annotated, where candidates that best explain the experimentally observed spectra are ranked. This workflow provides two advantages. Metabolites within the candidate set are all biologically relevant. Consequently, all computing times will be used to evaluate biologically relevant candidates. Although there is now a growing collection of annotated genome sequences (e.g., KEGG database [28], MetaCyc [29], and BiGG [30]) and tools for the reconstruction of genome-scale metabolic models (GEMs) [31,32], the completeness of these models is not guaranteed. GEM models are typically constructed using sequencing and annotation [33,34]. Significant experimental and computational efforts are required to augment the models on the basis of gene expression, proteomics, and metabolomics data [35]. Current models do not account for enzyme promiscuity, where an enzyme transforms alternate substrates in addition to its natural substrate, as defined by a reference metabolic model and as catalogued in organism databases [36,37,38,39]. As a result, defining the candidate set only on the basis of metabolites within the metabolic models naturally limits annotation. 

Selecting the candidate set from a large database can potentially enhance annotation by increasing the number of measured masses that have a match in the candidate set (Figure 1B). This database-based workflow first identifies candidate metabolites by querying one or more specified compound databases for all molecules whose exact masses match the experimentally measured masses detected in the sample. The resulting candidate set is then annotated and ranked as in the workflow, as shown in Figure 1A. As the size of the candidate set is large in comparison to the one in the model-based workflow, the annotation runtime increases, and so does the chance of annotation. As annotation accuracy is low, some measurements, however, may be annotated with biologically irrelevant identities, such as phytochemicals or drug compounds that cannot possibly accumulate in a mammalian cell culture. The end user could then sift through the ranked candidate metabolites to select biologically relevant candidates. This manual curation is time-consuming and relies on the user’s judgment. Using a metabolic model to filter the ranked candidate metabolites can facilitate this process. However, this results in the same discovery-related limitations as the workflow shown in Figure 1A, while also incurring a large computational cost. Importantly, not all the computational cost is necessary. It is highly unlikely that every compound in candidate sets derived from a large database is biologically relevant. Using manually curated metabolite databases such as KEGG to derive the candidate set is an attractive option, as the size of the candidate set is reduced when compared to using a large structural database. However, not all biologically relevant compounds are catalogued in such databases.

Our novel annotation workflow (Figure 1C), EMMF, applies an EMM-based filter to identify the candidate set. To create this model, we adopted a previously described method, PROXIMAL [40] (Supplementary Methods). Although originally developed to analyze the products of xenobiotic transformation reactions catalyzed by possible via cytochrome P450 (CYP) enzymes, PROXIMAL was shown to be also effective in predicting promiscuous enzyme products for *Escherichia coli* [41]. From reactant-product pair(s) (RPAIR) of an enzymatic reaction [42], PROXIMAL identifies a molecular pattern that transforms the reactant into product. Each pattern is associated with a reaction center and its first and second-level neighboring atoms. If a substrate of interest matches a pattern, then the corresponding operator is applied to generate a product, which we call a “derivative” metabolite. The EMM for a system of interest is generated using PROXIMAL by applying the operators generated from the enzymatic reactions encoded in the system’s genome(s) to all of metabolites already associated with the system on the basis of the enzymes’ reaction definitions. This step generates a set of derivative metabolites. The calculated exact masses of derivative metabolites are then used to filter the measured masses. If a derivative has a mass that matches a measured mass, then the SMILES string [43] of this derivative is searched against a chemical structure database (PubChem) to determine if it has been cataloged with a chemical name and identifier. The masses of metabolites in the reference metabolic model are also matched against the measured masses (as in Figure 1A). The union of matched derivatives and reference model metabolites constitute a biologically relevant candidate set. This candidate set is then used for annotation and the candidates are ranked, as in prior workflows. Pseudo-code for the EMMF workflow is provided in the Supplementary Methods. 

## 3. Results

### 3.1. Datasets, Reference Metabolic Models, and EMMs 

We compared the annotation workflows in Figure 1 by analyzing untargeted LC–MS data collected on samples from two different biological systems (Table 1, column group A). One set of LC–MS experiments were performed on samples from Chinese hamster ovary (CHO) cell cultures grown in a chemically defined medium. The second set of experiments was performed on samples from anaerobic cultures of bacteria collected from murine cecum. Each set of LC–MS experiments comprised two or more different methods. By treating the datasets independently, we were able to explore the influence of sample source and instrument method on EMMF’s performance. Details for the culture and LC–MS experiments are provided in the Supplementary Methods. The processed data were arranged into feature tables, where each feature was specified by a chromatographic retention time (RT), measured mass (*m/z*), and a set of associated product ion (fragment) masses and their relative intensities, that is, the MS/MS signature. The reference metabolic models for CHO cell and murine cecal microbiota were derived from genomes in the KEGG database. For the CHO cell, we obtained lists of metabolites and reactions cataloged in KEGG that are associated with the organism code *cge*. The cecal culture is a consortium of many species. We assembled a community-level model based on the taxonomic groups detected in the culture using a previously described procedure [44]. The numbers of reactions, metabolites, and unique masses in the two reference models are listed in Table 1 (column groups B). 

The EMM for each sample was generated using biotransformation operators derived from each model (Table 1, column group C). EMMs augment a metabolic model to include molecules that are not originally part of the metabolic model. This augmentation increases the number of unique masses within the model. The number of biologically relevant molecules in the candidate set thus significantly increased (Table 1, column group D) when compared to the number of metabolites in the reference metabolic model (57× and 72× for CHO cell and the gut microbiota, respectively). Similarly, the number of unique masses in EMM was increased over the number of unique masses in the reference metabolic model (23× and 30x for the CHO cell and the gut microbiota, respectively). EMMs thus promise to provide a large annotation space when compared to the reference metabolic model.

### 3.2. Annotation Opportunities

Compared to using the reference metabolic model for a biological sample, using an EMM as the search space during metabolite annotation increased the size of the candidate set for annotation in terms of (a) matching to a larger number of measured masses, and (b) suggesting a larger set of putative chemical identities. Using these two metrics, we compared the size of the “biologically relevant candidate sets” in the model- and EMMF-based workflows and compared that with the size of the candidate set using PubChem (Table 2). A small percentage of the measured masses were matched to masses of metabolites in the metabolic model. On average, 3.31% of measured masses could be potentially annotated using the metabolic model only. When using the EMMs, this number increased to 23.92%, a 7.6-fold increase. When restricting the EMM derivatives to those that had a catalog entry in PubChem, the annotation rate dropped to 5.12%, as there are many compounds that are not yet catalogued in PubChem, currently the largest structural database. Using PubChem, the number of mass matches are in the millions. Not all such metabolites are biologically relevant. The use of reference metabolic models or large databases such as PubChem therefore provide some limitations in annotation when compared to using EMMs. Using EMMF allows for novel biological discovery by suggesting biologically relevant compounds not in PubChem, and reduces the annotation space considerably. 

**Figure 2:**
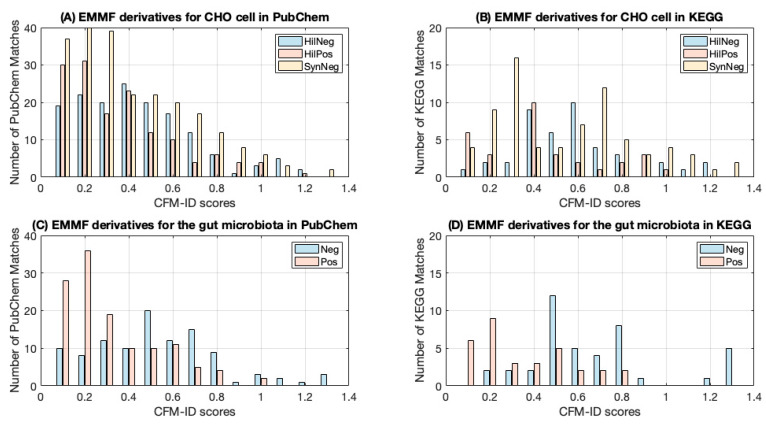
Distribution of CFM-ID scores for EMMF derivatives. (**A**) Chinese hamster ovary (CHO) cell derivatives that had a match in PubChem. (**B**) CHO cell derivatives that had a match in KEGG. (**C**) Gut microbiota derivatives that had a match in PubChem. (**D**) Gut microbiota derivatives that had a match in KEGG.

The quality of the EMMF candidates with known PubChem or KEGG identities (Supplementary Listing) were evaluated by using CFM-ID. The number of EMMF candidates that were associated with a KEGG or PubChem identities and the percentage of candidates that were associated with non-zero CFM-ID scores are shown in Table 3. On average across all datasets, 50% of annotations suggested by EMMF had a non-zero CFM-ID score. A considerable number of candidates received high CFM-ID scores, with an average CFM-ID score of 0.475 and 0.396 for KEGG and PubChem matches, respectively. The mean CFM-ID scores for the PubChem matches were lower than those for the KEGG matches. The distribution of the CFM-ID scores for the matches in PubChem and in KEGG varied (Figure 2). Lower scores may have indicated substructure matches corresponding to specific peaks. 

As the KEGG database is largely a small subset of PubChem, using all of the KEGG compounds as a candidate set for annotation may not be as computationally prohibitive as using PubChem. Further, using only a biological database such as KEGG for annotation guarantees biological relevance of candidate metabolites. A question that often arises regards the benefits of utilizing a general database for annotation compared to when employing a database that mostly comprises biomolecules. Using the EMMF workflow as a reference and restricting derivatives to those with chemical identities in PubChem, we were able to explore and quantify the benefits. Specifically, we utilized the EMMF workflow to identify candidate sets for our datasets. We then compared the EMM candidates against those obtained using the database-based workflows using KEGG and PubChem (Table 4). Many candidate molecules identified by EMMF that are catalogued in KEGG (e.g., for CHO cell HilNeg data, 93 out of 174 candidate compounds). However, there were also EMMF candidate compounds found in PubChem that were not catalogued in KEGG. For our datasets and using EMM metabolites as a reference, there was at least 2x or more additional biologically relevant candidates in PubChem for each candidate identified in KEGG. The twofold increase over KEGG is a lower bound on the number of biologically relevant metabolites in PubChem. Using a large database such as PubChem thus significantly increases biologically relevant annotation opportunities when compared to KEGG. Relying only on small biological databases limits annotation. EMMF provides an alternative candidate set that provides different tradeoffs between annotation opportunities and speed. 

The table reports the number of EMMF derivatives, percentage of EMMF derivatives that had non-zero CFM-ID scores, and the average score. These results are reported for EMMF candidates that had a matching identity in the KEGG and for PubChem databases.

### 3.3. Computational Time Required for Annotation 

To generate the candidate set as the input to in silico annotation analysis in database-based workflow (Figure 1B), we identified metabolites in the KEGG and PubChem databases that mass-matched within 10 ppm to the masses in our experimental data for each dataset. We investigated the computational time required to annotate the candidate sets from EMMs and from the combined PubChem and KEGG databases. Annotation of each candidate set identified by EMMF required a handful of hours, averaging 2.5 h per dataset (Table S1, group A). The number of candidate metabolites from databases PubChem and KEGG for each of our datasets exceeded 5 million candidates, with an average dataset size of 7.8 million candidates (Table S1, group B). It was computationally prohibitive to annotate all mass-matched metabolites from the databases. To calculate the required runtime, we estimated it using annotation runtimes based on the EMMF workflow (Table S1, group A). Dividing the runtime by number of metabolites in the candidate set, on average, annotation requires 0.0085 h per match. Using this average, the estimated runtime for annotation of database-based workflow was computed for each dataset. The average required runtime per dataset was over 65,000 h (Table S1, group B). 

### 3.4. Experimental Validation of EMMF

We next investigated whether any of the derivatives predicted by EMMF and matched to a detected MS feature based on mass and MS/MS signature could be experimentally confirmed with a chemical standard. We selected eight predicted derivatives that matched an LC–MS feature for CHO cell samples (Table 5, group A). The selection was based on two factors: the rank assigned by the in silico annotation tool and availability from a vendor. The selected derivatives were salicylaldehyde, one of the three isomers of hydroxybenzaldehyde; 4-hydroxyphenyllactate, a tyrosine metabolite; acetoacetamide, a monocarboxylic acid amide of acetoacetic acid; 5-aminopentanoate, a lysine degradation product; glutarate, produced in lysine and tryptophan metabolism; 3-methoxyanthranilate, an ester of anthranilic acid; 2-hydroxyphenylacetic acid, associated with styrene degradation pathway; and 4-pyridoxate, a product of vitamin B_6_. When using KEGG as a database for annotation, CFM-ID ranked six of the eight derivatives as the highest ranked candidates, whereas two of the derivatives were not in KEGG (Table 5, group B). Further, a small number of candidate matches were found for each mass measurement. When using PubChem as a database for annotation, all derivatives ranked among the three top candidates (Table 5, group C). As expected, the number of putative matches increased when compared to the number of matches using KEGG. The CFM-ID score for each candidate is provided in Table 6. The CFM-ID scores ranged from 0.596 for the spectral signature annotated by EMMF as salicylal, to 0.979 for the spectral signature annotated by EMMF annotated as 5-aminopentanoate. We analyzed the number of reactions in CHO that contributed an operator that was used to generate each derivative and the number of Enzyme Commission (E.C.) numbers that were associated with each set of reactions (Table 5, group D). The number of reactions and enzymes varied for each derivative. For example, 12 different reactions catalyzed by 15 enzymes corresponded to the operator that generated 4-hydroxyphenyllactate, whereas only one reaction and enzyme corresponded to the operator that generated acetoacetamide. 

**Figure 3:**
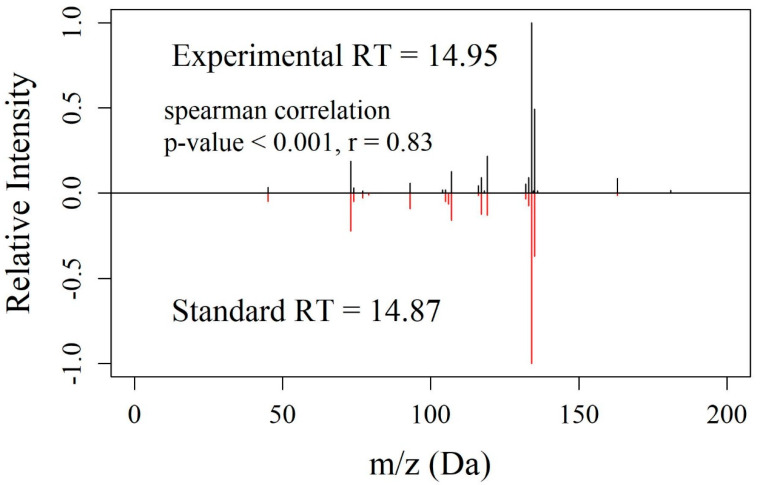
Mirror plot for 4-hydroxyphenyllactate, KEGG compound C03672. (**A**) Experimental data collected using untargeted metabolomics from the CHO cell culture. (**B**) Data from high-purity chemical standard. This is considered a match by retention time (RT; difference < 3 min) and by MS/MS (spearman rank correlation *p*-value < 0.05 and *r*-value > 0.6).

We compared the RTs and MS/MS spectra of standards for these chemicals against the corresponding CHO cell culture sample features (Supplementary Methods). We were able to confirm correct annotation of 4-hydroxyphenyllactate (Figure 3). This demonstrated that the EMMF can indeed identify a novel metabolite that was not found among metabolites cataloged for the organism of interest, in this case the Chinese hamster. 

In addition to KEGG, we searched for 4-hydroxyphenyllactate in MetaCyc. Neither database associated this metabolite with the Chinese hamster. In KEGG, 4-hydroxyphenyllactate is associated with three enzymatic reactions. Reactions 4-hydroxyphenyllactate:NAD+ oxidoreductase (Reaction R03336 in the KEGG database) and 4-hydroxyphenyllactate:NADP+ oxidoreductase (R03338) are both catalyzed by D-hydrogenase (E.C. 1.1.1.222, which was recently deleted and transferred to E.C. 1.1.1.110) and hydroxyphenylpyruvate reductase (E.C. 1.1.1.237). Reaction 3-(4-hydroxyphenyl)lactate hydro-lyase (4-coumarate-forming) (Reaction R08766 in the KEGG database) is associated with an enzyme that has yet to be characterized (E.C. 4.2.1.-). It is unlikely that the source of 4-hydroxyphenyllactate in our sample was exogenous, as our cell culture medium was chemically defined and did not include this metabolite. Further evidence that the metabolite is endogenously derived was provided by a recently updated genome-scale metabolic model (GEM) for the CHO cell in the BiGG database [30]. This GEM reconstruction (iCHOv1) included 4-hydroxyphenyllactate as a “universal” metabolite that could be formed enzymatically, but for which a specific gene encoding the enzyme remains unknown. None of the other standards confirmed matches (Figure S3). Further, upon careful examination of the spectral signature annotated as glutarate, we realized that the spectral signature was incorrectly selected via peak picking. We therefore excluded it from further analysis.

We further analyzed the spectral signatures comprising our experimental validation set using the Global Natural Products Social Molecular Networking (GNPS) spectral library [45]. There were suggested matches in GNPS (Table 6, column C). The spectral signature that was annotated by EMMF as acetoacetamide was matched in the GNPS library with aminocyclopropane and L-threonine (cosine scores 0.92 and 0.9, respectively). Neither of these candidates were suggested by PROXIMAL. The spectral signature that was annotated by EMMF as 2-hydroxyphenylacetic acid was matched in the GNPS library with 4-hydroxyphenylacetic acid (cosine score of 0.81). Compounds 2-hydroxyphenylacetic acid and 4-hydroxyphenylacetic acid are isomers that only differ in their hydroxyl group positions. The spectral signature that was annotated by EMMF as 4-pyridoxic acid was matched with the same compound in the GNPS library (cosine score 0.76). None of the other features matched to a metabolite in the GNPS library, including the metabolite that was identified by EMMF and experimentally validated as 4-hydroxyphenyllactate. 

We further explored the annotation of the experimental validation set using the Human Metabolome Database (HMDB) [46]. There were several suggested matches for four metabolites (Table 6, column D). The scores, however, were relatively low for all compounds. Homovanillic acid, with a score of 0.43, was incorrectly suggested as a match for the spectral signature experimentally verified as 4-hydroxyphenyllactate.

We report the number of PubChem compounds that matched in mass to the measured spectra (Table 6, column E), and the MetFrag analysis of the test spectra (Table 6, group F) using PubChem as a database. The number of candidates ranged from 241 to 1962. None of the tested compounds were ranked highly amongst the candidates, including the compound experimentally verified as 4-hydroxyphenyllactate. The rank for 4-hydroxyphenyllactate was 12. The top match via MetFrag for the compound annotated by EMMF as 2-hydroxyphenylacetic acid was methyl-phenyl-silyl-silane, a compound that includes silicon. There is a chance that this compound may be due to an unknown environmental contaminant from. Excluding this low-probability possibility, this compound is clearly not native to CHO cell metabolism, thus emphasizing the need for biologically relevant filters when performing annotations. 

## 4. Discussion and Conclusions

Our EMMF workflow addressed the challenge of creating an annotation candidate set that is enzymatically relevant to the sample under study and that includes metabolites beyond what is already catalogued in reference metabolic models. One important contribution of the work is conceptually separating the engineering of the candidate set from annotation, as EMMF creates a biologically relevant candidate set that can be utilized for putative identification. Prior works provided limited engineering of candidate sets. These works focused on exclusions of particular elements, substructures, or compounds [47], or on inclusion sets [48]. Filtering PubChem compounds using PubMed Medical Subject Heading (MeSH) labels [49] can reduce the candidate set size by including only naturally occurring compounds that are biologically relevant (carbohydrates, lipids, etc.). However, only a tiny fraction (124,049 compounds) of PubChem compounds has MeSH labels. Filtering using current MeSH labels was reported to reduce a candidate set of 62,782 structures that match in mass to 3868 compounds in the GNPS dataset to only 36 compounds [21]. 

Results from comparing the three workflows emphasized the need for optimizing the engineering of candidate sets. We demonstrated for our two case studies that using candidate sets from large databases is computationally prohibitive, as others have also noted [21]. We also demonstrated that using a biological database such as KEGG yields a smaller candidate set when compared to using a large structural database. Continued and significant growth of biological databases such as HMDB [46], which allows not only for candidate retrieval but also for spectral searches, promises to improve annotation rates and reduce the uncatalogued unknowns that must be explored in novel ways, as suggested herein. We further demonstrated that using a reference metabolic model is inadequate, as only a very small percentage (3.31% on average) of measurements can be annotated. In this regard, EMMF contributes two key advances. First, filtering candidate chemicals using an EMM allows for the identification of novel metabolites that are missing from a GEM reconstruction. This advance addresses the need to enable discovery, which is inherently limited in the simpler approach of using a model comprising only known metabolites to filter the candidate chemicals or when using a small biological database, without incurring a prohibitive computational cost. Second, filtering the measurements through an EMM specific for the system of interest provides a biologically relevant and computationally feasible candidate set. This advance eliminates unnecessary and time-consuming computations on chemicals from large databases that are likely irrelevant to the system of interest. Not all biologically relevant candidates from a large database are in the EMM. This issue could be addressed by further expansion of the EMM candidate set by the repeated application of the biotransformation operators derived from the reference model to derived promiscuous products. 

EMMF relies on a reference metabolic model for annotation. Other recent studies have also exploited the metabolic network to enhance annotation. One method, iMet, suggests that neighboring metabolites within a metabolic network have similar MS/MS spectra and trains a classifier to predict if two spectra belong to neighboring metabolites [50]. The classifier is trained using MS/MS spectra from spectral databases and mass differences between reactant pairs from KEGG that are not specific to the biological sample. Another method, BioCAN creates a network based on measured features and assigns aggregate annotation scores based on spectral lookups and annotation tools [51]. Mummichog maps features to metabolic models, and performs statistical pathway and module enrichment [52]. There are also other studies that exploit putative biotransformation for annotation. In one method, the mass difference between a pair of features is matched against mass differences between substrate–product pairs of common metabolic conversions (oxygenation, acetylation, etc.), with a match indicating a potential biochemical transformation between the pair of detected feature masses [53]. These transformations can be used to propagate metabolite annotation from an identified metabolite to its potential reactants and products. In contrast to this method, EMMF does not require any MS/MS training data and utilizes biological context that is specific to the sample to suggest a candidate set. There is a common limitation when using metabolic models to improve annotation. Genome-scale metabolic reconstructions can be incomplete, especially for non-model organisms. EMMF suggested that 4-hydroxyphenyllactate may result from the promiscuous activity of one or more carboxylic acid dehydrogenases expressed in the CHO cell on 4-hydroxyphenylpyruvate. Using a chemical standard, we confirmed the presence of 4-hydroxyphenyllactate in the CHO cell samples analyzed in this study, even though there is no documented gene associated with CHO cell metabolism that can catalyze the reaction with 4-hydroxyphenyllactate as product. Our result is supported by other recent papers that report on the presence of this metabolite in CHO cell cultures [54,55].

This work presents the first in vivo experimental evidence for a computationally predicted metabolite derived through promiscuous action of an enzyme. Using a chemical standard, we confirmed the presence of 4-hydroxyphenyllactate in a CHO cell culture, even though there is no documented gene associated with the CHO cell metabolism that can catalyze the reaction with 4-hydroxyphenyllactate as product. We were, however, able to confirm only one out of the eight predicted metabolites. This could be due to inaccuracies in the rankings by the annotation tools. Analyses of the tested compounds using the GNPS spectra database, HMDB, CFM-ID, and MetFrag showed significant variations in the annotation results. The low confirmation rate can also be due to the assumption that all enzymes are promiscuous. As an enhancement, we are currently investigating methods to improve *PROXIMAL* to rank predicted derivatives on the basis of enzyme designations as generalists or specialists [56] and participation in primary or secondary metabolism [57]. The current version of PROXIMAL is available through the web portal http://hassounlab.cs.tufts.edu/proximal. This work did not evaluate the quality of candidates that did not have a match in PubChem or KEGG. A thorough evaluation of these candidates may have yielded biologically relevant matches. 

It is possible to utilize other tools or databases to identify metabolites that could occur due to enzyme promiscuity. For example, BioTransfomer utilizes a knowledgebase (MetXBioDB) and a reasoning engine to predict enzyme products [58]. MetXBioDB provides chemical and biological information for deriving biotransformation rules that can be utilized with the reasoning engine. The BioTransfomer metabolite identification tool analyzes biotransformations associated with human, gut microbiome, or environmental enzymes to suggest promiscuous enzyme products for an input molecule. Similarly, the MINEs database [59] extends other databases of known metabolites by computing new structures that follow a set of biochemical transformation rules [60]. The MINEs database was incorporated in MS-FINDER 2.0 to support an annotation function that retrieves structural isomers of predicted formulas for a given spectral signature [61]. In contrast to Biotransformer and MINEs, which use curated generic biotransformation rules, PROXIMAL utilizes organism-specific transformation rules derived specifically from reactions within the organism’s metabolic model. A systematic evaluation of such promiscuity prediction tools can shed light on the tradeoffs between a limited number of generic but highly curated rules vs. a larger number of automatically generated rules when predicting promiscuous products.

Despite limitations due to the underlying potentially incomplete metabolic models and to the accuracy of current annotation tools, EMMF demonstrates utility in creating an expanded, biologically relevant candidate set and in utilizing it to enhance annotation. This utility is demonstrated via the discovery of 4-hydroxyphenylpyruvate and in high annotation scores using CFM-ID for some EMMF derivatives. Importantly, EMMF promises to offer annotation opportunities beyond those possible with metabolic models without the high computational cost of searching large structural databases that contain many non-biological compounds. 

## Figures and Tables

**Table 1:** Size of experimental data sets and models. (A) Three experimental datasets under different conditions were collected for the CHO cell, and two for the gut microbiota sample. (B) The size of the metabolic model: number of reactions, metabolites, and unique masses. (C) The size of the expanded metabolic model: number of operators derived using *PROXIMAL*, unique derivatives generated by *PROXIMAL*, unique derivative masses due to *PROXIMAL*. For comparison purposes, the numbers of derivatives and derivative masses exclude those in the metabolic model. (D) Fold increase in number of metabolites and masses when comparing the size of these sets for EMM against the metabolic model.

	(A) Experimental Data			(B) Metabolic Model			(C) Expanded Metabolic Model Using PROXIMAL			(D) Fold Change for EMM Relative to Metabolic Model	
Biological Sample	Dataset	MS Mode	Number of Measured Masses	Number of Reactions	Number of Metabolites	Number of Unique Masses	Number of Unique Operators	Number of Unique Derivatives	Number of Unique Derivative Masses	Number of Metabolites	Number of Unique Masses
CHO cell	HilNeg	negative	2502	1619	1353	775	2392	76745	17930	56.72	23.14
	HilPos	positive	3856								
	SynNeg	negative	5336								
gut microbiota	Neg	negative	1651	1381	1307	779	2756	94186	23356	72.06	29.98
	Pos	positive	1657								

**Table 2:** Candidate set size using different workflows. (A) Candidate set size when using the model: number of measured masses that match to metabolites in the model, the equivalent percentage of the number of measured masses reported for experimental data in Table 1, and corresponding number of chemical identities. (B) Candidate set size when using extended metabolic model (EMM)-based filtering: number of measured masses that match to metabolites in the EMM, equivalent percentage in reference to the number of measured masses reported for experimental data in Table 1, and corresponding number of chemical identities. (C) Further filtering of the EMM derivatives reported in column group (B) to include only mass measurements that match to previously known chemical IDs as reported in PubChem, and reporting the number of matched masses, the relative percentage of these masses to the number of measured masses reported for experimental data in Table 1, and the corresponding number of chemical IDs. (D) Size of the candidate set when filtering using PubChem.

Biological Sample		(A) Metabolites in Metabolic Model			(B) All EMM Derivatives			(C) EMM Derivatives with Previously Known Chemical IDs			(D) Using PubChem-based Filtering	
		Number of Measured Masses Matched to Those in Metabolic Model	Percentage of Measured Masses Matched to Those in Metabolic Model	Number of Chemical Ids Associated with Measured Masses	Number of Masses Matched to Those in EMM	Percentage of Masses Matched to Those in EMM	Number of Unique Mass-Matched Derivatives in EMM But Not in The Model	Number of Masses Matched to Those with Previously Known Chemical IDs	Percentage of Masses Matched to Those with Previously Known Chemical IDs	Number of Previously Known Chemical IDs for EMM Derivatives that Mass-Match to Measurements	Number of Unique Mass Matches in PubChem	Number of Corresponding Chemical IDs Associated with Measured Masses
CHO cell	HilNeg	118	4.72%	178	678	27.10%	2,725	174	6.95%	386	3,951,635	7,657,564
	HilPos	75	1.95%	93	715	18.54%	2,729	132	3.42%	226	3,362,305	6,406,877
	SynNeg	198	3.71%	229	1,490	27.92%	4,944	293	5.49%	527	7,058,696	14,133,885
gut microbiota	Neg	51	3.09%	131	445	26.95%	2,470	77	4.66%	207	2,448,238	5,192,205
	Pos	36	2.17%	43	316	19.07%	1,236	84	5.07%	149	2,774,074	5,572,587
Averages		96	3.13%	135	729	23.92%	2,821	152	5.12%	299	3,918,990	7,792,624

**Table 3:** Percentage of EMMF candidates with non-zero CFM-ID scores and their average scores.

Biological Sample		KEGG			PubChem		
		Number of EMMF Derivatives	Percentage of EMMF Derivatives with Nonzero CFM-ID scores	Average CFM-ID Score	Number of EMMF Derivatives	Percentage of EMMF Derivatives with Nonzero CFM-ID scores	Average CFM-ID Score
CHO cell	HilNeg	65	65%	0.557	280	55%	0.415
	HilPos	48	63%	0.395	286	49%	0.316
	SynNeg	114	64%	0.501	446	51%	0.370
gut microbiota	Neg	252	16%	0.631	197	53%	0.484
	Pos	56	55%	0.292	428	29%	0.270
Average			53%	0.475		47%	0.396

**Table 4:** Using EMMs to compare the annotation opportunities of PubChem against the KEGG database.(A) Experimental data for different datasets (repeated for convenience). (B) Number of matched masses and candidate chemicals found using EMMF that are reported in KEGG. (C) Number of matched masses and candidate chemicals found using EMMF reported in PubChem but not in KEGG. (D) Lower-bounds on discovery of biologically relevant matched masses and candidate chemicals when using PubChem over KEGG.

Biological Sample	(A) Experimental Data		(B) In EMM And in KEGG		(C) In EMM And PubChem, And Not in KEGG		(D) Lower-Bound Fold Increase of Pubchem over KEGG	
	Dataset	Number of Measured Masses	Number of Matched Masses	Number of Candidate Chemical IDs	Number of Matched Masses	Number of Candidate Chemical IDs	Number of Matched Masses	Number of Candidate Chemical IDs
CHO cell	HilNeg	2502	56	93	118	200	2.11	2.15
	HilPos	3856	26	39	106	148	4.08	3.79
	SynNeg	5336	88	122	205	283	2.33	2.32
gut microbiota	Neg	1651	25	47	52	113	2.08	2.40
	Pos	1657	23	28	61	93	2.65	3.32
Average							2.65	2.80

**Table 5:** Candidate metabolites identified by EMMF that were used for experimental validation. (A) Candidate mass and name. (B) Ranking of metabolite and number of candidates that matched mass measurement using KEGG. (C) Ranking of metabolite and number of candidates that matched mass measurement using PubChem. (D) The number of reactions that yielded the *PROXIMAL* operator that yielded each candidate metabolite and the associated number of enzymes that catalyze these reactions. (E) The status of experimental validation.

(A) Candidate Metabolites		(B) KEGG		(C) PubChem		(D) PROXIMAL		(E)
Mass Measurement (Daltons)	Candidate Metabolite Identified by EMMF	Rank	Matches	Rank	Matches	Number of Reactions Used to Derive Operator	Number of ECs Associated with Reactions	Experimentally Validated?
122.04	Salicylaldehyde	1	1	1	1	1	1	No
182.06	4-Hydroxyphenyllactate	1	2	1	4	12	15	Yes
101.05	Acetoacetamide	1	1	2	3	1	1	No
117.79	5-Aminopentanoate	1	2	1	5	4	4	No
132.04	Glutarate	1	1	3	6	12	11	No
167.06	3-Methoxyanthranilate	1	1	2	3	8	2	No
152.05	2-Hydroxyphenylacetic acid	NA	1	1	4	1	1	No
183.05	4-Pyridoxate	NA	0	1	1	1	1	No

**Table 6:** EMMF candidate metabolites analyzed using annotation tools and databases.(A) Candidate metabolite suggested by EMMF on the basis of scores from CFM-ID. (B) CFM-ID score. (C) Name of top match compound(s) and its score based on the GNPS spectral library. (D) Name of top match compounds and its score based on HMDB. (E) Number of PubChem candidates based on a 10ppm window of the measured mass. (F) MetFrag results, including the rank of the compound identified via EMMF based on CFM-ID scores and compound availability, its associated number of peaks in the spectral signature that MetFrag explained compared to the number of peaks that were utilized to provide the MetFrag ranking, the top match provided by MetFrag, and its associated number of peaks in the spectral signature that MetFrag explained compared to the number of peaks that were utilized to provide the MetFrag ranking.

	(A) EMMF	(B) CFMID	(C) GNPS	(D) HMDB	(E) PubChem	(F) MetFrag			
Mass Measurement (Daltons)	Candidate Metabolite	Score	Matched Compound ( Score)	Matched Compound (Score)	Number of Matches	Rank of Compound Identified by EMMF	# of Peaks Explained/ # of Peaks Used	Top Ranked Candidate	# of Peaks Explained/ # of Peaks Used
122.04	Salicylal	0.596	No Match	No Match	241	27	4/8	2-cyclopenta-1,3-dien-1-yl-2-oxo-acetaldehyde	4/8
182.06	4-Hydroxyphenyllactate	0.717	No Match	Homovanillic acid (0.43)	1694	218	10/22	methyl 2-hydroxy-2-phenyl-peroxyacetate	11/22
101.05	Acetoacetamide	0.682	Aminocyclopropane (0.92), L-threonine (0.90)	No Match	445	331	1/2	hydroxy N-isopropenylmethanimidate	1/2
117.79	5-Aminopentanoate	0.979	No Match	L-Valine (0.44), Betaine (0.34), 5-Aminopentanoic acid (0.31)	858	12	2/5	2-[ethyl(methyl)amino]acetic acid	2/5
132.04	Glutarate	0.600	No Match	Ethylmalonic acid (0.41)		N/A			
167.06	3-Methoxyanthranilate	0.949	No Match	Mandelic acid (0.55), 3-Hydroxyphenylacetic acid (0.44), p-Hydroxyphenylacetic acid (0.40), Ortho-Hydroxyphenylacetic acid (0.19)	1962	972	2/7	(2-aminophenyl) peroxyacetate	2/7
152.05	2-Hydroxyphenylacetic acid	0.716	4-hydroxyphenylacetic acid (0.81)	No Match	841	129	1/4	methyl-phenyl-silyl-silane	1/4
183.05	4-Pyridoxate	0.870	4-Pyridoxate (0.76)	No Match	1252	149	2/5	2-[1-(3-furyl)ethylideneamino]oxyacetic acid	2/5

## Supplementary Materials

The following are available online at https://www.mdpi.com/2218-1989/10/4/160/s1, Supplementary Methods is a PDF file that provides a detailed description of PROXIMAL and EMMF. Supplementary Listing is an excel spreadsheet that lists promiscuous enzymatic products in SMILES format for the CHO and gut microbiota samples.
